# Nuclear Drosha enhances cell invasion via an EGFR-ERK1/2-MMP7 signaling pathway induced by dysregulated miRNA-622/197 and their targets *LAMC2* and *CD82* in gastric cancer

**DOI:** 10.1038/cddis.2017.5

**Published:** 2017-03-02

**Authors:** Liyun Xu, Yixuan Hou, Gang Tu, Yanlin Chen, Yan-e Du, Hailong Zhang, Siyang Wen, Xi Tang, Jiali Yin, Lei Lang, Kexin Sun, Guanglun Yang, Xiaoli Tang, Manran Liu

**Affiliations:** 1 Key Laboratory of Laboratory Medical Diagnostics, Chinese Ministry of Education, Chongqing Medical University, Chongqing 400016, China; 2 Experimental Teaching Center of Basic Medicine Science, Chongqing Medical University, Chongqing 400016, China; 3 Department of Endocrine and Breast Surgery, The First Affiliated Hospital of Chongqing Medical University, Chongqing 400016, China

## Abstract

Drosha is an RNA III-like enzyme that has an aberrant expression in some tumors. Our previous studies showed the aberrant Drosha in gastric tumors. However, the roles of nuclear *Drosha*, the main regulator of microRNA (miRNA) biogenesis, in gastric cancer (GC) progression remain poorly understood. In this study, we demonstrated that nuclear Drosha is significantly associated with cell invasion of GC and that *Drosha* silence impedes the tumor invasion. Knockdown of *Drosha* led to a set of dysregulated miRNAs in GC cells. Multiple targets of these miRNAs were the members in cell migration, invasion and metastasis-associated signaling (e.g. ECM-receptor interaction, focal adhesion, p53 signaling and MAPK signaling pathway) revealed by bioinformatics analysis. *LAMC2* (a key element of ECM-receptor signaling) and CD82 (a suppressor of p53 signaling) are the targets of miR-622 and miR-197, respectively. High levels of LAMC2 and low levels of *CD82* were significantly related to the worse outcome for GC patients. Furthermore, overexpression of *LAMC2* and knockdown of CD82 markedly promoted GC cell invasion and activated EGFR/ERK1/2-MMP7 signaling via upregulation of the expression of phosphorylated (p)-EGFR, p-ERK1/2 and MMP7. Our findings suggest that nuclear Drosha potentially has a role in the development of GC.

Gastric cancer (GC) is the most common gastrointestinal cancer with high morbidity and mortality in China. There are ~740 000 deaths each year, accounting for 10% of total cancer death.^1^ Tumor invasion and metastasis are the major cause for the high mortality rate of GC. Recently, multiple molecular alterations, such as the activation and overexpression of oncogenic *RAS*,^2^ inactivation of tumor suppressor genes *p53*^3^ and even some of the noncoding RNA^4^ have been shown to be involved in gastric carcinoma metastasis. However, the mechanism of gastric tumor metastasis is not fully understood.

Drosha is an enzyme of endonuclease RNase III, which is critical for the canonical microRNA (miRNAs) biogenesis. Aberrant expressions of Drosha are closely related to carcinogenesis and cancer progression. However, there are controversial reports about Drosha expressions in different human malignancies. For example, the downregulated Drosha proteins were detected in gallbladder cancer and the decreased Drosha was used as indicators of poor prognosis in gallbladder cancer patients.^5^ In contrast, the increased Drosha expressions were observed in non-small-cell lung cancer,^6^ which was closely related to the pathological stage, tumor metastasis and worse prognosis. It is inseparable that the role of aberrant *Drosha* in the tumor progression may be dependent on miRNAs, which have referred to tumor initiation and development as oncogenes or tumor suppressor genes through negative regulation of hundreds of target genes at the post-transcriptional level.^7^ Dysregulated miRNAs are detected in different kinds of cancers, including colorectal carcinoma,^8^ breast cancer^9^ and GC,^4^ and involved in tumor pathology, diagnosis, treatment, prognosis and other processes. For example, miR-21 inhibits lung squamous carcinoma cell proliferation and metastasis by targeting *PTEN* and *RECK*.^10^

Our previous studies have shown that the aberrant Drosha expression may be associated with tumor malignancy in GC.^11^ However, the roles of Drosha in human GC remains to be elucidated. In this study, we further analyzed *Drosha* expression in gastric tumor tissues and their adjacent normal tissues by immunohistochemistry (IHC) and qRT-PCR. The silence of Drosha expression using interfering RNA in GC led to impeded tumor cell invasion and change of miRNA profiles. Knockdown of *Drosha* significantly reduced cell invasion via an EGFR-ERK1/2-MMP7 signaling pathway, which is partly due to miR-622 and miR-197 targeting *LAMC2* and *CD82*, respectively. Thus, our study provides a mechanistic insight into the function of *Drosha* in gastric metastasis via an altered miRNA profile.

## Results

### Drosha expression in GC tissues and cell lines

**Figure 1:**
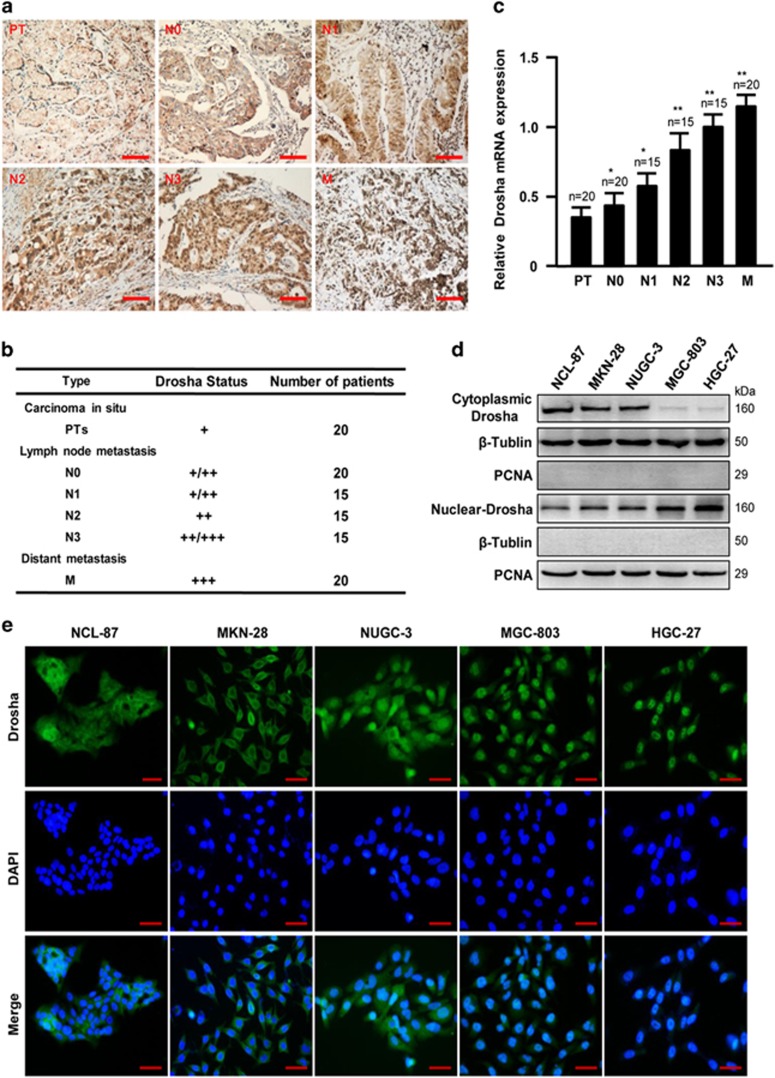
Drosha expression in GC tissues and cell lines. (**a**) Representative images of Drosha staining in the tumor *in situ* (PT), lymph node metastasis tissues (N0–N3) and distant metastasis tissues (M). Scale bars, 100 *μ*m. (**b**) Summary of IHC staining for carcinoma *in situ* (PT), lymph node metastasis (N0–N3) and distant metastasis (M) GC samples. The nuclear staining intensities were categorized as low (+), medium (++) or high (+++) based on observations of 80% of the cell population. (**c**) Quantitation of *Drosha* mRNA was measured by qRT-PCR. The data represent means±S.D. from triplicate samples (*P<0.05, **P<0.01 *versus* PT). (**d**) Cytoplasmic and nuclear Drosha levels were determined by western blot in indicated GC cells. *β*-Tubulin: cytoplasmic protein marker; PCNA: nuclear marker. (**e**) Expression and localization of Drosha by IF staining in GC cells. Scale bars, 50 *μ*m

Our previous studies have shown that aberrant nuclear Drosha was upregulated in GC.^11^ To understand whether high levels of nuclear Drosha are a bad predictor for patients with GC, we further detected Drosha expression in gastric tumor tissues by IHC staining. Consistent with our previous findings, the nuclear Drosha was significantly higher in gastric adenocarcinoma than that in the tumor *in situ* (preinvasive tumor, PT) and normal gastric tissues (data are not shown). Compared with PTs, the gradually enhanced nuclear *Drosha* proteins were detected in lymph node metastasis tissues (N0–N3) and distant metastasis tissues (M) (Figures 1a and b). The similar mRNA expression patterns of Drosha were disclosed in these tissues (Figure 1c). To further verify this association of the Drosha expression pattern and the malignancy of GC, the expressions and distribution of Drosha were assessed in four of the poorly differentiated GC cells (MKN-28, NUGC-3, BGC-803 and HGC-27) and the well-differentiated GC cell (NCL-87) by western blot and immunofluorescence (IF) staining; as expected, the enhanced nuclear Drosha was observed in malignant GC cells (Figures 1d and e). These data indicate that the high levels of nuclear Drosha may associate with GC metastasis.

### *Drosha* silence reduces cell migration potential of GC cells

**Figure 2:**
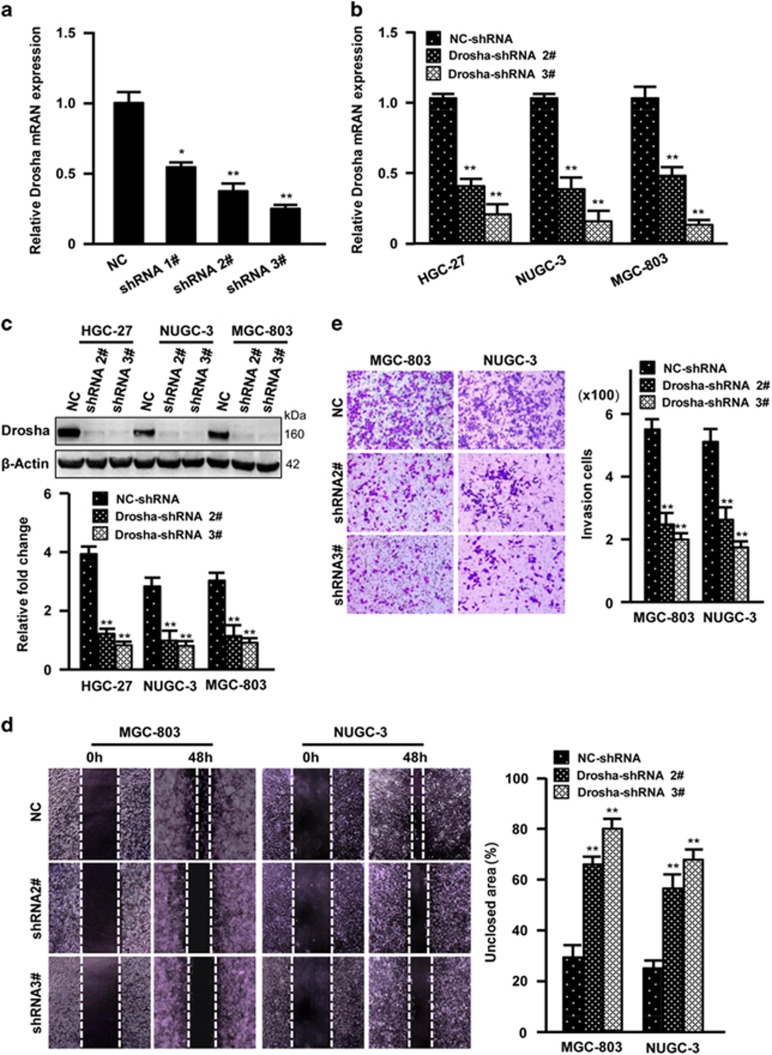
*Drosha* silence inhibits GC cell invasion. (**a**) The efficiency of short hairpin RNAs (shRNAs) against *Drosha* in 293T cells was determined by qRT-PCR (*P<0.05, **P<0.01 *versus* control shRNA). (**b** and **c**) Interference of Drosha in HGC-27, NUGC-3 and MGC-803 GC cells was detected by qRT-PCR (**b**) and western blotting (**c**) (**P<0.01 *versus* control shRNA). *β*-Actin works as a loading control. (**d** and **e**) Cell mobility and invasion were tested by wound healing assay (**d**) or Transwell assay (**e**) for indicated GC cells with specific shRNA (2# or 3#) against *Drosha*. The mobility ability was determined as the percentage of non-healed scratched area by using the Image J software. The data are presented as the mean±S.D. (*n*=3; **P<0.01 *versus* control shRNA)

To further understand the roles of Drosha in GC metastasis, siRNA interference of *Drosha* expression was used. *Drosha* was verified to be efficiently knocked down by shRNA against *Drosha* in MGC-803 GC cells (Figure 2a). Thus, the lentivirus-mediated shRNA 2# and shRNA 3# were stably infected into GC MGC-803, NUGC-3 and HGC-27 cells. Efficiency of knocking down *Drosha* (Figures 2b and c) led to a clear slowdown of motility ability (Figure 2d) and invasive potentials of MGC-803 and NUGC-3 cells (Figure 2e). These data suggest that *Drosha* may have a role in promoting migration and invasion of GC cells.

### miRNA profiles were dysregulated in *Drosha*-silenced GC cells

**Figure 3:**
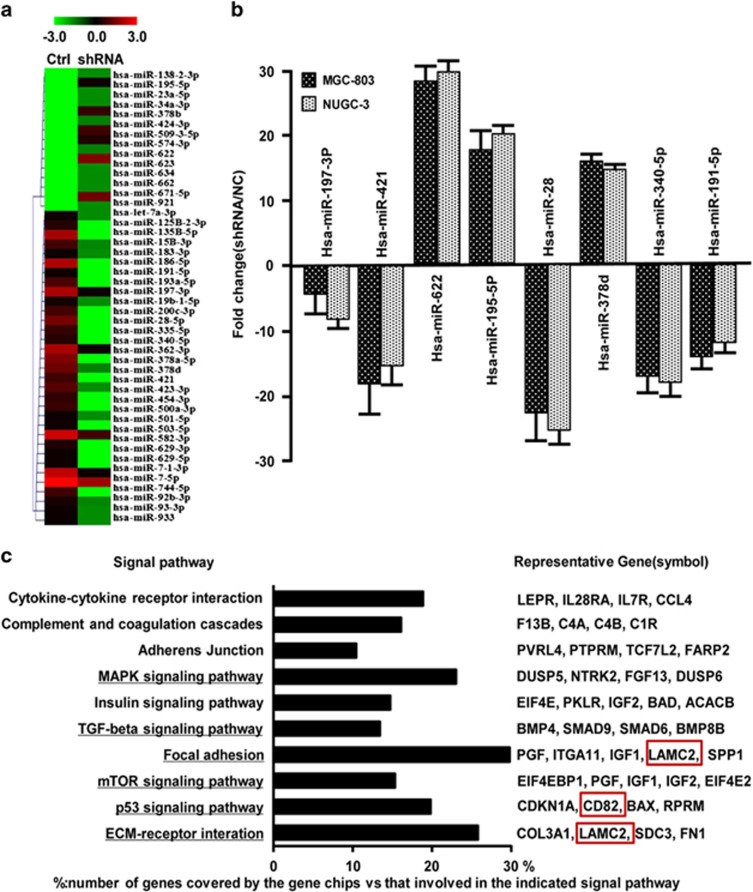
Global changes of miRNA profile in *Drosha*-silenced GC cells. (**a**) The heatmap of 47 altered miRNAs identified by miRNA array in *Drosha*-knockdown cells. (**b**) qRT-PCR to certify the altered miRNAs for the cumulative microarray data. (**c**) The major changed pathways (*P*<0.05) of copredicted miRNA targets were enriched by DAVID v.6.7. The black column represents the number of copredicted miRNA target genes located on the pathway. The representative target genes and the interesting targets were shown on the right panel. Percentage (%)=the target genes/the total genes in a given pathway

It has been known that *Drosha* has an important role in the canonical miRNA biogenesis in the nucleus. Thus, we guessed that some of the miRNAs and their target genes associated with cell migration and invasion may respond to dysregulated *Drosha* in the GC. Indeed, a set of miRNAs (47 upregulated and 14 downregulated) were identified in *Drosha*-knockdown MGC-803 cells by miRNA array analysis (Figure 3a). To validate the miRNA assay data, eight of the randomly chosen dysregulated miRNAs were tested by qRT-PCR in MGC-803 and NUGC-3 cells (Figure 3b). Using TargetScan v.6.2, miRanda and DIANA-microT, a total of 4088 subsequent function mRNA targets was predicted. The significantly altered signaling pathways (*P*<0.05) were compiled using DAVID v.6.7, in which ECM-receptor signaling pathway, p53 signaling pathway, focal adhesion signaling, MAPK signaling pathway, TGF-β signaling pathway and mTOR signaling pathway were reported to be related to cell migration, invasion and metastasis (Figure 3c).

### miR-622 and miR-197, respectively, direct target *LAMC2* and *CD82*

**Figure 4:**
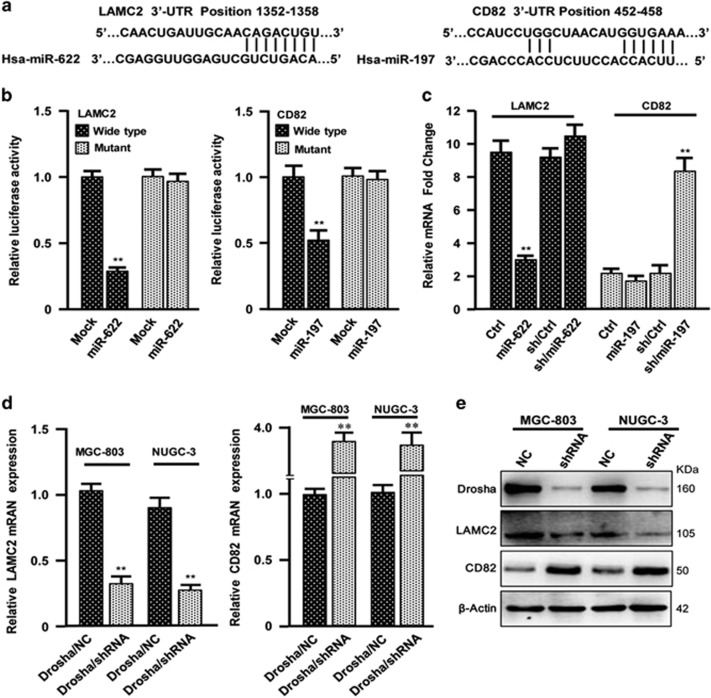
*LAMC2* is a direct target of miR-622 and *CD82* is a target of miR-197. (**a**) The predicted binding sites of miR-622 and miR-197 in *LAMC2* and *CD82* 3′-UTRs. (**b**) Luciferase activities of *LAMC2* and *CD82* were tested in MGC-803 cells co-transfected with miR-622 or miR-197 mimics and their mimics control with wild-type (WT)/Mut 3′-UTR of *LAMC2* (left panel) or *CD82* (right panel). The mean±S.D. represents three independent experiments (**P<0.01 *versus* mock). (**c**) qRT-PCR to test the endogenous mRNAs of *LAMC2* and *CD82* in the indicated cells (**P<0.01 *versus* control). (**d** and **e**) The indicated mRNAs (**d**) and proteins (**e**) were analyzed by qRT-PCR and western blotting assay in Drosha WT and silenced MGC-803 and NUGC-3 cells. *β*-Actin is the loading control (**P<0.01 *versus* control shRNA cells)

Next, we wondered which key miRNAs and their associated targets or signaling pathways may have critical roles in GC metastasis. Twenty of the top changed miRNAs in our miRNA array, their functional targets and corresponding signaling pathways were carefully analyzed by bioinformatics or paper reviewing. The *LAMC2* and *CD82* were found to be closely related with ECM-receptor signaling pathway or p53 signaling pathway, which was suggested to contribute to tumor metastasis. Six of these miRNAs, including miR-622, miR-197, miR-199b-5p, miR-146a, miR-129 and miR-130-5p, may be the regulators of *LAMC2* and *CD82*. Of these, miR-622 and miR-197 have a high score to bind to the 3′-UTR of *LAMC2* or *CD82*, respectively (Figure 4a). These were further verified by luciferase assay. *LAMC2* was suppressed by miR-622 and *CD82* was repressed by miR-197. In addition, mutation of the binding sites in the 3′-UTRs of *LAMC2* or *CD82* canceled the responsiveness of these genes to ectopic miR-622 or miR-197 in MGC-803 cells (Figure 4b). Similarly, the endogenous mRNA levels of *LAMC2* in MGC-803 cells were significantly decreased under overexpression of miR-622. The levels of *LAMC2* had no significant change when miR-622 was knocked down by its specific shRNA (Figure 4c, left panel). On the other hand, there was no much change of endogenous *CD82* after it was transfected with miR-197 in MGC-803 cells. However, the mRNA of *CD82* was markedly enhanced after knockdown of miR-197 using shRNA in MGC-803 cells (Figure 4c, right panel). Knocking down Drosha in MGC-803 and NUGC-3 cells, the decreased LAMC2 or increased CD82 was detected in mRNA and protein levels (Figures 4d and e). These data indicate that *LAMC2* is a major target of miR-622, and *CD82*, a major target of miR-197.

### Aberrant LAMC2 and CD82 expressions are associated with bad outcome for GC patients

**Figure 5:**
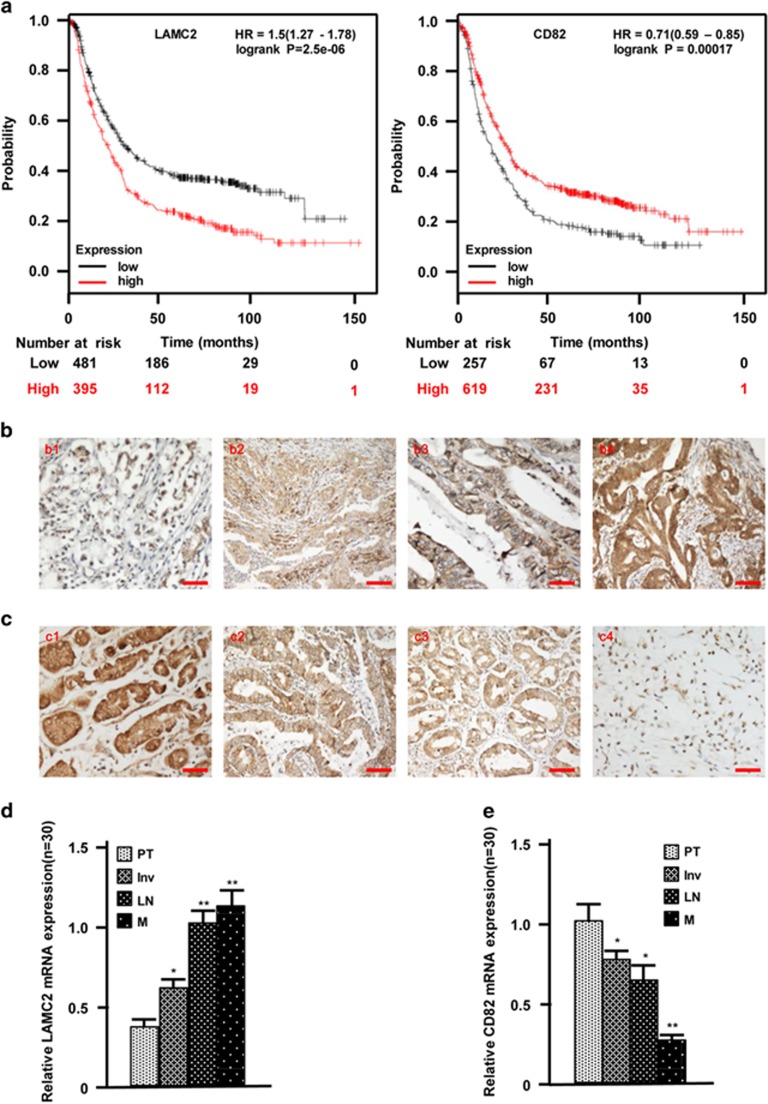
The enhanced LAMC2 and decreased CD82 indicate tumor metastasis and worse outcome of GC. (**a**) The overall survival of GC patients was evaluated by Kaplan–Meier plots. (**b**) The gradual increase of LAMC2 proteins were examined by IHC staining in GC specimens from the tumor *in situ* (b1), invasive tumor tissues (b2), tumors with lymph node metastasis (b3) and metastasis tissues (b4). (**c**) IHC staining to show the gradual decrease of CD82 proteins in GC tissues derived from the tumor *in situ* (c1), invasive tumors (c2), tumors with lymph node metastasis (c3) and tumors with distant metastasis locus (c4). (**d** and **e**) The expressions of LAMC2 (**d**) and CD82 (**e**) in various gastric carcinomas were quantitatively determined by qRT-PCR. PT: tumor *in situ*; Inv: invasive tumor; LN: tumor with lymph node metastasis; M: tumor with distant metastasis locus (*P<0.05, *P<0.01, *versus* PT). Scale bars, 100 *μ*m

To characterize the association of LAMC2 and CD82 with the prognosis of GC, the Kaplan–Meier survival curves were applied using 876 GC patients in the KM plots database (http: //www.kmplot.com), and GC patients with higher levels of LAMC2 and/or lower levels of CD82 had longer survival (Figure 5a). Consistently, higher levels of LAMC2 and lower levels of CD82 had a significant effect on cell invasion and metastasis of GC revealed by IHC staining (Figures 5b and c, *P*<0.01) and qRT-PCR analysis (Figures 5d and e, *P*<0.01). Taken together, these data demonstrate that higher levels of LAMC2 and lower levels of CD82 were significantly associated with GC invasion and metastasis.

### LAMC2 promotes and CD82 suppresses GC invasion via an EGFR/ERK1/2-MMP7 signaling pathway

**Figure 6:**
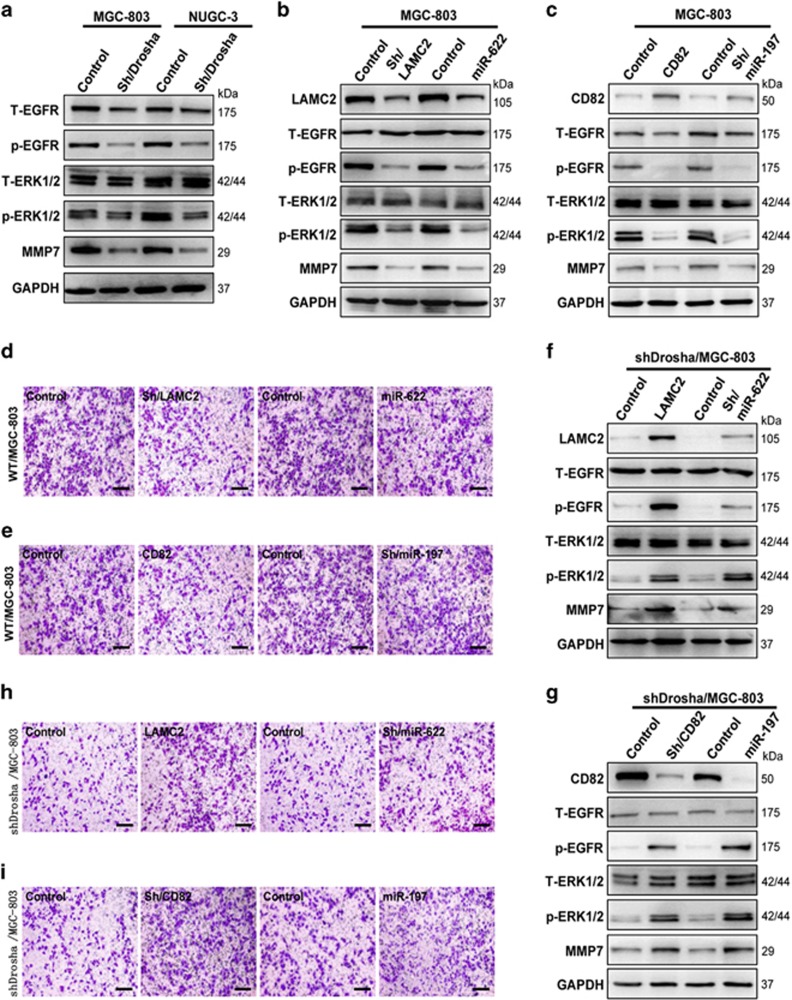
The enhanced LAMC2 orchestrates with decreased CD82 to promote tumor cell invasion via an EGFR-ERK1/2-MMP7 pathway. (**a**) The activated EGFR and ERK1/2 were detected in Drosha wild-type (WT) MGC-803 and NUGC-3 cells by western blotting. (**b**) The decreased p-EGFR and inactivation of ERK1/2 and lower levels of MMP7 were determined by western blotting in LAMC2-knockdown or miR-622-overexpressing MGC-803 cells. (**c**) Rescue of CD82 or knockdown of miR-197 in MGC-803 cells, and western blotting was used to determine the indicated protein expressions. (**d** and **e**) Cell invasion of MGC-803, as in (**b**) and (**d**), was tested using Transwell chambers. (**f**) Rescuing LAMC2 expression or stably knocking down miR-622 expression in Drosha-silenced MGC-803 cells, p-EGFR, p-ERK1/2 and MMP7 were determined by western blotting. (**g**) Western blotting to detect the indicated protein expressions in the MGC-803 cells with Drosha knockdown. (**h** and **i**) The cell-invasive potential was tested using the Transwell chamber for Drosha-silenced MGC-803 cells, as in (**f**) and (**g**). Glyceraldehyde 3-phosphate dehydrogenase (GAPDH) worked as a loading control in all western blotting analysis experiments

LAMC2 could colocalize with EGFR in the ATC cells^12^ and CD82 suppresses the phosphorylation of EGFR in EGF- and HGF-dependent manner in Hepa1–6 cells,^13^ indicating that the aberrant LAMC2 and CD82 may be involved in tumor cell invasion through the activity of EGFR and its downstream signaling in GC cells. Indeed, the activities of EGFR and its downstream ERK1/2 signaling were repressed, and expression of MMP7 was decreased in Drosha-silenced MGC-803 and NUGC-3 cells (Figure 6a). To further confirm these findings, the specific shRNA against *LAMC2* or the miR-622 mimics was stably or transiently transfected into MGC-803 cells. The activities of EGFR and ERK1/2 were suppressed, and MMP7 expression was reduced after *LAMC2* interfered (Figure 6b). Correspondingly, tumor cell-invasive potentials were decreased in these cells (Figure 6d). The similar results were observed after rescuing *CD82* expression by stably transfecting pcDNA-*CD82* or sh/miR-197 into MGC-803 cells (Figures 6c and e). Furthermore, the restoration of LAMC expression in *Drosha*-silenced MGC-803 cells led to the enhanced phosphorylation of EGFR and ERK1/2, and high levels of MMP7 (Figure 6f). As a result, the potential of tumor cell invasion was increased (Figure 6h). In line with this, knockdown of *CD82* expression by sh/*CD82* or miR-197 in *Drosha*-silenced MGC-803 cells resulted in the increased expression of p-EGFR, p-ERK1/2 and MMP7 (Figure 6g), which rendered the tumor cells with a marked invasive potential (Figure 6i). These data suggest that the EGFR/ERK1/2-MMP7 signaling pathway is the core in LAMC2-promoting and CD82-suppressing GC cell invasion.

## Discussion

Drosha is a nuclear enzyme that cleaves the primary miRNAs to precursor miRNAs in the classic miRNA biosynthesis.^14^ Our previous studies have shown that the aberrant nuclear Drosha may be associated with tumor malignancy and differentiation in GC.^11^ However, the biological function of Drosha in GC was still unclear. Here we disclosed that the nuclear *Drosha* promotes tumor cell invasion in malignant GC cells through aberrant miRNA biosynthesis, and thus regulates their targets and corresponding signaling pathways.

Aberrant Drosha expressions have even been reported to closely associate with some solid tumor progression. Interestingly, the increased Drosha was usually related to pathologic features of the tumor, metastasis and prognosis in non-small-cell lung cancer.^6^ However, the enhanced nuclear Drosha was inversely correlated with tumor grade (breast cancer)^15^ or tumor invasion (e.g. human cutaneous melanoma),^16^ suggesting that the different function of Drosha may be based on different tumor types.

We found that nuclear Drosha has a key role in GC cell invasion. Higher levels of nuclear Drosha were detected in invasive and metastatic gastric carcinoma and cancer cells. Knockdown of Drosha expression decreased tumor cell invasion in the detected GC cells with higher nuclear Drosha. Using miRNA microarray and bioinformatics, most of the miRNA targets that were found to be the key members belonged to signaling pathways, which are associated with cell invasion and cancer metastasis, such as ECM-receptor signaling pathway, p53 signaling pathway and MAPK signaling pathway. Our works support that abnormal expression of nuclear Drosha was critical to tumor metastasis-related miRNA generation as previous findings in cervical SCC cells^17^ and Drosha cKO mice.^18^

miRNA-622 and miRNA-197 expressions were dysregulated and were closely related to tumor cell invasion in GCs. Using bioinformational and experimental evidence, we confirmed that *LAMC2* was a miR-622 target, and CD82, a miR-197 target. *LAMC2* (laminin *γ*2) is the only monomeric chain that was secreted from laminin 332, consisting of the *α*3, *β*3 and *γ*2 chains. Higher levels of LAMC2 are in most of the malignant tumors and correlate with poor overall survival and metastasis, as a therapeutic target for human cancers,^19, 20^ whereas *CD82* (also called *KAI*-1), as a metastasis suppressor gene, is downregulated in cancers.^21^ These data support our findings that enhanced LAMC2 and decreased CD82 in GC cells are critical for gastric tumor invasion and metastasis.

Activation of EGFR-ERK1/2-MMP7 signaling axis regulated by nuclear Drosha may have a key role in GC cell invasion. EGFR and its downstream signaling have a critical function in gastric carcinoma development, and targeting EGFR may be a major strategy in the personalized treatment of gastrointestinal tumors.^22^ The amplified EGFR is a worse factor in GC initiation, invasion and metastasis.^23^ LAMC2 colocalizes with EGFR to phosphorylate EGFR and activates its downstream signaling ERK1/2.^12, 24^ CD82 suppresses EGFR expression and phosphorylation in EGF- and HGF-dependent manner in Hepa1–6 cells.^15^ Here we observed that the high level of nuclear Drosha decreased miR-622 or increased miR-197 to lead to an upregulated LAMC2 and downregulated CD82 in malignant GC cells. Knockdown of *LAMC2* (or rescue of miR-622) expression or knockdown of miR-197 (or rescue of CD82) expression in MGC-803 cells inhibited tumor cell invasion. Inversely, the cell-invasive potential could be partially restored in *Drosha*-silenced MGC-830 cells. However, the reason why *Drosha* processes miR-622 and miR-197 differently is still unknown.

**Figure 7:**
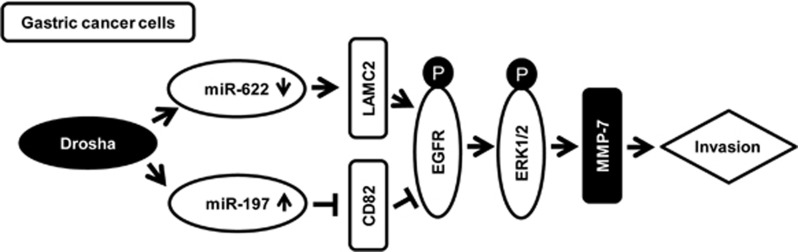
A model depicts the role of Drosha promoting GC cell invasion. Drosha activates EGFR-ERK1/2 signaling to promote GC cell invasion via regulation of miR-197, miR-622 and their targets LAMC2 and CD82

In summary, the current study reveals that nuclear *Drosha* may involve in the biogenesis of a set of metastasis-related miRNAs in GCs. Higher levels of miR-197 via downregulation of *CD82* in coordination with lower levels of miR-622 via upregulation *LAMC2* in GC activate EGFR-ERK1/2 signaling (Figure 7), thus having a role in promoting tumor cell invasion and metastasis.

## Materials and methods

### Tissue specimens

Human gastric tumor tissues and their corresponding precancerous tissue were obtained from patients with gastric tumor undergoing surgery at the first affiliated hospital of Chongqing Medical University. None of the patients had previously undergone radiotherapy or chemotherapy treatment. All tissues included 20 normal adjacent tissue samples, 20 tissues derived from the tumor *in situ*, 65 lymph node metastasis tissue samples (N0=20, N1=15, N2=15 and N3=15) and 20 distant metastasis tumor tissues (M). The investigation was approved by the ethics committee of the Chongqing Medical University.

### Cell culture

Human GC cell lines MKN-28 and HGC-27 and human normal gastric epithelial cell line GES-1 were obtained from the Shanghai Cell Bank of the Chinese Academy of Sciences (Shanghai, China). MGC-803, NUGC-3 and NCL-87 cell lines were kindly donated by Prof. Yang Ke of the Beijing Institute of Cancer Research (Beijing Shi, China). MGC-803 and GES-1 were maintained in DMEM containing 10% FBS (Gibco-BRL, Australia); NUGC-3, HGC-27, MKN-28 and NCL-87 were routinely cultured in RPMI-1640 (Gibco) with 10% FBS. All cells were incubated in a humidified atmosphere of 5% CO_2_ at 37 °C.

### Plasmid construction, inhibitors and mimics

The pHBLV-*LAMC2* and CFP-*CD82* plasmids were purchased from Hanbio Biotechnology Co. (Shanghai, China) or Addgene Inc. (http://www.addgene.org/). All the synthetic shRNA oligonucleotides used in this study were obtained from GenePharma (Shanghai, China). The transfection of shRNAs was performed using the Lipofectamine 2000 reagent (Invitrogen, Carlsbad, CA, USA). The pMIR-*LAMC2*- and pMIR-*CD82*-Report vector were obtained by inserting wild-type and mutant binding sites of miR-622 or miR-197 in the 3′-UTR of LAMC2 or CD82 into the pMIR-Report vector (Ambion, Austin, TX, USA) at the *Spe*I and *Hind*III sites. Mimics and inhibitors of miR-622, miR-197 and their controls were purchased from GenePharma (Shanghai, China). The sequences described above are provided in Supplementary Table 1.

### Immunofluorescence

IF assay was performed as described previously.^25^ The primary antibody against Drosha (1:200; no. 12286; Abcam, Cambridge, MA, USA) or IgG control, and FITC-labeled goat anti-rabbit secondary antibody (1:200; Sigma, St. Louis, MO, USA) were used in IF. Cell nucleus was stained with DAPI. Immunofluorescent images were captured using a Nikon Eclipse 80i microscope (Eclipse 80i, Tokyo, Japan).

### Immunohistochemistry

Immunohistochemical staining was performed as described previously.^26^ Briefly, the deparaffinized tissue sections (4 *μ*m) were heated for antigen retrieval, quenched for endogenous peroxidase activity and blocked with goat serum; the primary antibodies against Drosha (1:150; ab12286; Abcam), LAMC2 (1:150; Millipore, Darmstadt, Germany), CD82 (1:150; CST, Danvers, MA, USA), and secondary antibody (1:100; ZSBIO, Beijing, China) were used. After staining with diaminobenzidine and hematoxylin, the images were captured and assessed by the Image-Pro plus 6.0 software (Media Cybernetics, Rockville, MD, USA) to quantify the immunohistochemical staining. The mean optical density in 50 randomly selected areas (MOD=IOD/area) was used to evaluate the levels of protein expression.

The pathology scoring of the tissues was performed as described previously.^26^ Nuclear expression of Drosha was graded from 1+ to 3+ (1+, 1–25% 2+, 26–50% 3+ and >50% nuclear staining). The staining intensities of Drosha were assessed by examining 80% of the cell population. Images were captured using a Nikon Eclipse 80i microscope (Eclipse 80i).

### RNA extraction and qRT-PCR

Total RNA was isolated using TRIzol (Invitrogen) routinely. The quantitative real-time PCR was performed in triplicate using SYBR Premix Ex Taq II (RR820A; TaKaRa, Dalian, China). Relative gene expression was measured as 2^Ct(internal control)^^−Ct(gene)^. The primers used are listed in Supplementary Table 2.

### Western blot analysis

Nuclear and cytoplasmic extracts were prepared using a Nuclear and Cytoplasmic Protein Extraction Kit (Beyotime Institute of Biotechnology, Jiangsu, China) as described previously.^11^ Total proteins were extracted using RIPA lysis buffer. Fifty micrograms of cell lysates were electrophoresed with 10% SDS-PAGE. The specific primary antibodies used against each protein in the immunoblotting were as follows: anti-Drosha (1:1000; Abcam), anti-PCNA (1:800; Abcam), anti-LAMC2 (1:1000; Millipore), anti-CD82 (1:1000; CST); the antibodies anti-EGFR, anti-p-EGFR, anti-ERK1/2, anti-p-ERK1/2, anti-MMP7 and anti-GAPDH (1:1000; all from Beyotime); anti-*β*-tubulin (1:500; Santa Cruz Biotechnology, Santa Cruz, TX, USA) and anti-*β*-actin (1:1000; Boster, Wuhan, China). The appropriate horseradish peroxidase-conjugated secondary antibodies were subsequently applied. The proteins were visualized by the enhanced chemiluminescence system (Amersham, Pharmacia Biotech, Freiburg, Germany).

### Wound healing and cell invasion assays

The experimental procedures of wound healing assays and cell invasion assay was described in detail previously.^25^ The NUGC-3 and MGC-803 cells were used in these assays. The width of scratches was recorded by phase contrast microscopy (Nikon TE2000-U; Nikon Corporation, Tokyo, Japan) and was measured using the Image J software (Rawak Software, Inc, Germany) at the designated time point. The percentage of the non-healed scratched area (S) for each replicate was calculated as follows: % of the non-healed scratched area=(S (designed time)/S (starting time)) × 100%. The invaded cells for invasion assay were counted in five of the randomly selected fields. The data represent three experiments, each in triplicate (mean±S.E.).

### miRNA array assay and bioinformatics analysis

The miRNA array assay was performed as described previously in detail.^27^ The MGC-803/*Drosha*-shRNA and MGC-803/NC-shRNA control cells were used in the Agilent miRNA array (Agilent, Santa Clara, CA, USA). The raw data were normalized by Quantile algorithm using the Gene Spring Software12.6 (Agilent, Santa Clara, CA, USA). These miRNAs were then grouped using hierarchical clustering with ‘complete' using Heatmap.2 function (gplots package v.2.9.0).

The potential target genes for the dysregulation miRNAs were predicted using TargetScan v.6.2 algorithms, miRanda algorithms and DIANA-microT algorithms software (WWW.microrna.gr/WebServer) as described previously.^27^ All the copredicted target genes were used for subsequent functional analysis through DAVID Bioinformatics Resources 6.7. The *P*-value cutoff was set below 0.05.

### Luciferase reporter assay

Luciferase assays were performed as described previously.^28^ MGC-803 cells were co-transfected with a total 800 ng of miR-622, miR-197 expression plasmid or its control vector and the pMIR-*LAMC2*, pMIR-*CD82* report vector (wild-type or mutant) or control vector using Lipofectamine 2000 (Invitrogen). pRL-TK worked as an internal control. The *Renilla* and firefly luciferase activities were measured by a Dual-Luciferase Reporter System (E1910; Promega, Madison, WI, USA) according to the manufacturer's instructions.

### Statistical analysis

Statistical analysis was performed using the SPSS standard version 13.0 software (Chicago, IL, USA). The data of three independent experiments were presented as the mean±S.D. The independent Student's *t*-test was used to compare the continuous variables between two groups. A *P*-value <0.05 was considered to be statistically significant.
