# Supplementary material for: Nuclear Drosha enhances cell invasion via an EGFR-ERK1/2-MMP7 signaling pathway induced by dysregulated miRNA-622/197 and their targets LAMC2 and CD82 in gastric cancer
Source: Cell Death Dis. 2017 Mar 2;8(3):e2642–. doi: 10.1038/cddis.2017.5 (PMC5386557; doi:10.1038/cddis.2017.5)
Supplement: Supplementary Tables [file cddis20175x1.pdf]

Supplementary Table1.
The siRNA/mimics sequences specifically against the target gene

Gene name	sequence
miR-197 shRNA	5’-GCUGGGUGGAGAAGGUGGUGAA-3’
miR-622 shRNA	5’-GCUCCAACCUCAGCAGACUGU-3’
Negative control	5’-CAGUACUUUUGUGUAGUACAA-3
CD82 shRNA	5’UCUCGAAUGAGCUCAGUCACGAUGC-3
LAMC2 shRNA	5’-GGUUCUCUUAGUGCUCGAUTT-3
Drosha shRNA 1#	5’-CGAGUAGGCUUCGUGACUUTT-3’
Drosha shRNA 2#	5’-ACGAAGCTCGATGAAGATTTA-3’
Drosha shRNA 3#	5’-AACGAGUAGGCUUCGUGACUU-3’
miR-197 mimics	5’-UUCACCACCUUCUCCACCCAGC-3’
miR-622 mimics	5’-ACAGUCUGCUGAGGUUGGAGC-3’
negative control	5’-UUCUCCGAACGUGUCACGUTT-3’

Supplementary Table2. Primers used for qRT-PCR analysis

Primer names	sequence
Drosha	F:CGATGATGCAGGGAAACACATG<br>R:TTATTTCTTGATGTCTTCAGTCT
LAMC2	F:AAGTCAGTACCAGAACCGAG<br>R:TTTCCCTTGTCAGTTGCTCC
CD82	F: TCAGCCTGTATCAAAGTCACC<br>R: CCCATGAGCATAGTGACTGCC
Actin	F:TGACGTGGACATCCGCAAAG<br>R:CTGGAAGGTGGACAGCGAGG
miR-197	F:CTGGAGTTCACCACCTTCTCCA<br>R: GTGCAGGGTCCGAGGT
miR-622	F:GAGGAAGTAAAAGGCTTACAA<br>R:GCTTGACCTTGATGTTCAGCAGG
miR-421	F:TATGGTTGTTCTGCTCTCTGTGTC<br>R:CTCACTCACATCAACAGACATTAATT
miR-28	F:TGGTAGACAACATGCTTGATGCTG<br>R:AGGCGGGATTGTAATCTAGGATTAACA
miR-195	F:CGTAGCAGCACAGAAATATTGGC<br>R:CCAGTCTCAGGGTCCGAGGTATTC
miR-378	F:CAGTGCGTGTCGTGGAG<br>R:AGCTAACACTGGACTTGGAGT
miR-191	F: ACACTCCAGCTGGGCAACGGAATCCCAAAA<br>R:CTCAACTGGTGTCGTGGA
miR-340	F: GCGCTAGGTAGTTTCCTGTT<br>R: GTGCAGGGTCCGAGGT
U6	F:TGCGGGTGCTCGCTTCGCAGC<br>R:CCAGTGCAGGGTCCGAGGT
